# Induction of Neutralizing Antibody Response against Four Dengue Viruses in Mice by Intramuscular Electroporation of Tetravalent DNA Vaccines

**DOI:** 10.1371/journal.pone.0092643

**Published:** 2014-06-02

**Authors:** Eakachai Prompetchara, Chutitorn Ketloy, Poonsook Keelapang, Nopporn Sittisombut, Kiat Ruxrungtham

**Affiliations:** 1 Dengue Vaccine Research Unit, Chula Vaccine Research Center (ChulaVRC), Faculty of Medicine, Chulalongkorn University, Bangkok, Thailand; 2 Department of Laboratory Medicine, Faculty of Medicine, Chulalongkorn University, Bangkok, Thailand; 3 Department of Microbiology, Faculty of Medicine, Chiang Mai University, Chiang Mai, Thailand; 4 Medical Biotechnology Unit, National Center for Genetic Engineering and Biotechnology, National Science and Technology Development Agency, Bangkok, Thailand; 5 Vaccine and Cellular Immunology Laboratory, Department of Medicine, Faculty of Medicine, Chulalongkorn University, Bangkok, Thailand; University of Rochester, United States of America

## Abstract

DNA vaccine against dengue is an interesting strategy for a prime/boost approach. This study evaluated neutralizing antibody (NAb) induction of a dengue tetravalent DNA (TDNA) vaccine candidate administered by intramuscular-electroporation (IM-EP) and the benefit of homologous TDNA boosting in mice. Consensus humanized pre-membrane (*prM*) and envelope (*E*) of each serotypes, based on isolates from year 1962–2003, were separately cloned into a pCMVkan expression vector. ICR mice, five-six per group were immunized for three times (2-week interval) with TDNA at 100 µg (group I; 25 µg/monovalent) or 10 µg (group II; 2.5 µg/monovalent). In group I, mice received an addtional TDNA boosting 13 weeks later. Plaque reduction neutralization tests (PRNT) were performed at 4 weeks post-last immunization. Both 100 µg and 10 µg doses of TDNA induced high NAb levels against all DENV serotypes. The median PRNT50 titers were comparable among four serotypes of DENV after TDNA immunization. Median PRNT50 titers ranged 240–320 in 100 µg and 160–240 in 10 µg groups (*p* = ns). A time course study of the 100 µg dose of TDNA showed detectable NAb at 2 weeks after the second injection. The NAb peaked at 4 weeks after the third injection then declined over time but remained detectable up to 13 weeks. An additional homologous TDNA boosting significantly enhanced the level of NAb from the nadir for at least ten-fold (*p*<0.05). Of interest, we have found that the use of more recent dengue viral strain for both vaccine immunogen design and neutralization assays is critical to avoid a mismatching outcome. In summary, this TDNA vaccine candidate induced good neutralizing antibody responses in mice; and the DNA/DNA prime/boost strategy is promising and warranted further evaluation in non-human primates.

## Introduction

Dengue virus (DENV) infection is a major public health problem in tropical and sub-tropical countries. Approximately 2.5 billion people are at risk and 50–100 million infections occur annually worldwide [1]. Unfortunately, licensed vaccine to prevent dengue is currently unavailable although various vaccine development strategies have been investigated [2]–[9]. As four antigenic-related serotypes of DENV (DENV-1, DENV-2, DENV-3 and DENV-4) commonly co-circulate, thus an effective vaccine must cover all serotypes. Safety, balance between immunogenicity and attenuation, and “interference” among DENV serotypes represent problems for live, attenuated dengue vaccine candidates [10]–[16]. DNA vaccine is an alternative strategy to overcome the concern of these problems together with its many advantages [17], [18]. In addition, while a live-attenuated dengue vaccine requires at least 6 months apart from the last immunization to be able to re-immunize as an effective boosting dose [19], [20]. DNA vaccine may have an advantage to be able to boost much sooner.

In this study, we constructed TDNA dengue vaccine candidates encoding the prM and E proteins with a very high homology DNA sequence to recent dengue viral isolates; and examined their immunogenicity following repeated IM-EP in mice. We also investigated whether an additional boosting with the homologous TDNA when the NAb was declining, a TDNA-prime/TDNA-boost strategy, would further enhance the neutralizing antibody responses.

## Materials and Methods

### Ethics statement

The animal experiments were performed according to the National Institutes of Health guidelines for care and use of laboratory animals. All experimental procedures were approved by the Committee of Animal Care and Use of Faculty of Medicine, Chulalongkorn University (approval no. 05/54). Immunization and bleeding procedures were performed under isoflurane-induced anesthesia.

### Cells and viruses

Monkey kidney-derived cell lines (Vero and LLC-MK_2_ cells, ATCC origin), DENV-1: strain 16007, DENV-2: strain 16681, DENV-3: strain 16562 and DENV-4: strain 1036 and C0036 were kind gifts from Drs. Ananda Nisalak and Robert Gibbons, Armed Forces Research Institute of Medical Sciences, Thailand. Vero and LLC-MK_2_ were propagated in minimum essential medium supplemented with 10% fetal bovine serum (FBS) and in Medium 199 supplemented with 20% FBS, respectively (all reagents were from Gibco) and incubated at 37°C, 5% CO_2_. Dengue viral stocks were propagated in Vero cells and stored in −80°C. Virus titers were determined by a plaque assay on LLC-MK_2_ cell monolayer.

### TDNA construction

**Figure 1:**
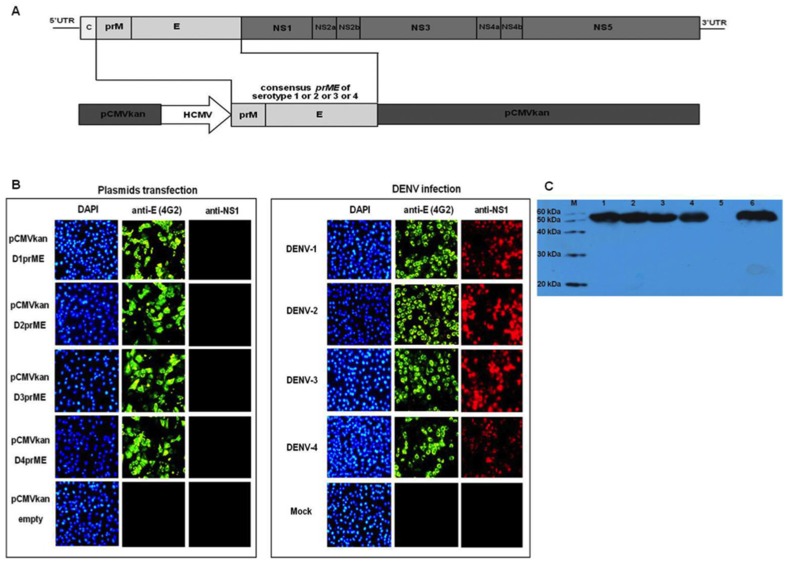
(A) Dengue DNA vaccine construction. Humanized sequence of the consensus *prME* genes from each dengue serotypes were cloned into pCMVkan expression vector. ss: signal sequence; HCMV: human CMV promoter. (B) Intracellular dengue proteins expression. Vero cells were transfected with indicated dengue DNA vaccine constructs or infected with DENV. The transfected or infected cells were stained with DAPI, anti-flaviviruses E mAb (4G2) or anti-DENV NS1 antibody, and analyzed using fluorescence microscopy. (C) Immunoblot analysis of secreted E protein. Cell culture supernatants were collected at 24 hr post transfection or infection, and analyzed by employing anti-flavivirus E antibody (clone 4G2). Lanes 1–5, recombinant plasmid pCMVkanD1, −2, −3, −4 prME and pCMVkan empty vector; lane 6, DENV-2 strain 16681. M: protein marker.

Full length humanized codon of consensus *prM/E* were generated from 133, 124, 54 and 65 dengue viral sequences of serotype 1, 2, 3 and 4, respectively which deposited in Genbank during 1962–2003 then commercially synthesized GeneArt (Germany). The Kozak sequence and *prM* signal sequence (capsid carboxy terminus) were inserted at the N-terminus. The expression cassettes were subcloned into pCMVkan expression vector [21] and designated as pCMVkanD1prME, pCMVkanD2prME, pCMVkanD3prME and pCMVkanD4prME (Figure 1A). All recombinant plasmid constructs were transformed into *Escherichia coli* DH5αF' (Invitrogen) and confirmed by nucleotides sequence analysis.

### Protein expression

Vero cells were separately transfected with individual recombinant plasmid constructs (pCMVkanD1prME-pCMVkanD4prME) using lipofectamine 2000 (Invitrogen). At 24 hr post-transfection, cells were fixed, permeabilized and stained with flavivirus-reactive anti-E antibody (clone 4G2) [22] and anti-DENV-NS1 antibody (clone DN3, Abcam). Rabbit-anti-mouse IgG-FITC (Dako) and goat-anti-mouse IgG-Alexa-fluor (Molecular Probe) were used as secondary Ab for detection of anti-E and anti-NS1, respectively. Cell nuclei were counter stained with 4, 6-diamino-2-phenylindole hydrochloride (DAPI) (Sigma–Aldrich). Stained cells were visualized under fluorescence microscope. Western blot was used for detection of E protein expression in cells culture supernatant at 24 hr post-transfection or infection by using 4G2 mAb. The cell culture supernatants (crude) were directly subjected for protein detection, transfected cells were not lysed before supernatant collection. Rabbit-anti-mouse IgG conjugated with horseradish peroxidase (KPL) was used as secondary Ab and detected by chemiluminescence substrate (Immobilon western, Millipore) then exposed to an X-ray film. Vero cells infected with DENV-2 (strain 16681) at the multiplicity of infection of 0.5 or transfected with empty pCMVkan expression vector were employed as positive and negative controls, respectively.

### Mice experiments

ICR mice at 4–6 weeks of age were procured from the National Laboratory Animal Center, Mahidol University, Thailand. Mice were immunized with DNA constructs by intramuscular *in vivo* electroporation, IM-EP (Ichor Medical Systems) at the tibialis muscle as previously described [23].

Five-six mice/group were immunized with TDNA cocktail at a total of 100 µg (25 µg of each the monovalent preparation) or 10 µg (2.5 µg each) per dose for 3 times at a 2-week interval using IM-EP. Mice were bled at 4 weeks after the last immunization and the sera were individually examined for NAb activity against each of the four dengue serotypes.

In the prime-boost study, six mice were immunized with 100 µg of the TDNA cocktail (25 µg of each the monovalent preparation) for 3 times at a 2-week interval and then boosted with 100 µg of the TDNA cocktail on week 17. Mice were bled at week 4, 6, 8, 10, 17 and 20 after the first immunization.

### Plaque reduction neutralization test (PRNT)

NAb titer was determined by PRNT as previously described [24]. Briefly, mice sera were inactivated at 56°C, 30 min and serially diluted with MEM supplemented with 10% FBS. Diluted sera were mixed with equal volume of target virus (30–50 PFU/well) and incubated at 37°C for 1 hr. Virus-serum mixture was transferred onto LLC-MK2 monolayer and allowed to absorb for 1 hr at room temperature. Cells were overlaid with first overlayer medium containing FBS, amino acid, vitamin, L-glutamine, 0.9% low-melting point agarose (Invitrogen), Hank's BSS and NaHCO_3_. After 4–5 days of incubation in 37°C, 5% CO_2_, the secondary overlayer containing 4% v/v neutral red (Sigma-Aldrich) was added. Plaques were counted after 24 hr of additional incubation. The highest serum dilution that resulted in 50% or more reduction of the average number of plaques as compared with the virus control wells was considered as the neutralizing endpoint titer (PRNT50).

### Statistic analysis

The comparisons of NAb (PRNT50) between experimental groups or at different time-points were performed with the Mann-Whitney test. *p*<0.05 was considered significant.

## Results

### *In vitro* protein expression analysis

At 24 hr post transfection, E protein, but not NS1, expression was detected in the cytoplasm of Vero cells transfected with each of the recombinant dengue prME DNA constructs (Figure 1B). Vero cells that were infected with dengue viruses showed both cytoplasmic E and NS1 protein expression. These two proteins were not detected in mock-infected Vero cells. Extracellular E protein, approximately 55 kDa in size, was detected after 24 hr post transfection in immunoblot analysis using the mAb 4G2 (Figure 1C) for all constructs, but not the empty expression vector.

### Induction of neutralizing antibody response in mice

**Figure 2:**
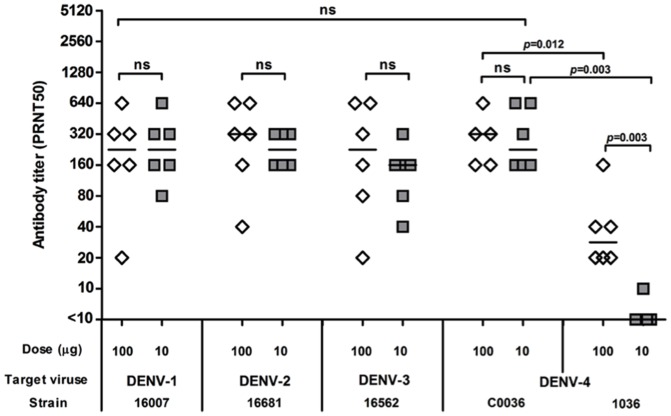
NAb responses against reference viruses used in PRNT in mice immunized with TDNA. NAb activities in sera of individual mouse following an IM-EP immunization with 100 µg/dose (opened diamond) and 10 µg/dose (grey square) for three times were determined at week 4 after the third dose. Horizontal lines represent the median PRNT50 titer for each group of mice (*n* = 5–6). ns: no statistically significant difference.

Mice immunized for three times with either 100 µg or 10 µg of total TDNA by IM-EP showed high levels of NAb against all four DENV serotypes. At 100 µg/dose of TDNA, the induced NAb titers against four dengue serotypes were comparable. The median PRNT50 titers against DENV-1, DENV-2, DENV-3 and DENV-4 (strain C0036) were 240, 320, 240 and 320, respectively. Slightly lower levels of NAb were detected in mice after three injections of 10 µg TDNA/dose. An interesting finding was observed during the PRNT testing of sera against DENV-4. When strain 1036 was used initially as target in the PRNT, the NAb titers were significantly lower than those of other serotypes. When a more recent isolate, C0036, was employed, the magnitude of NAb titers improved significantly; the median NAb titers were 320 and 240 in the 100 µg/dose and 10 µg/dose TDNA groups, respectively (Figure 2). A similar observation had been reported previously with recent clinical samples from DENV-4-infected individuals [25].

### Kinetics of NAb response following a prime-boost TDNA immunization

**Figure 3:**
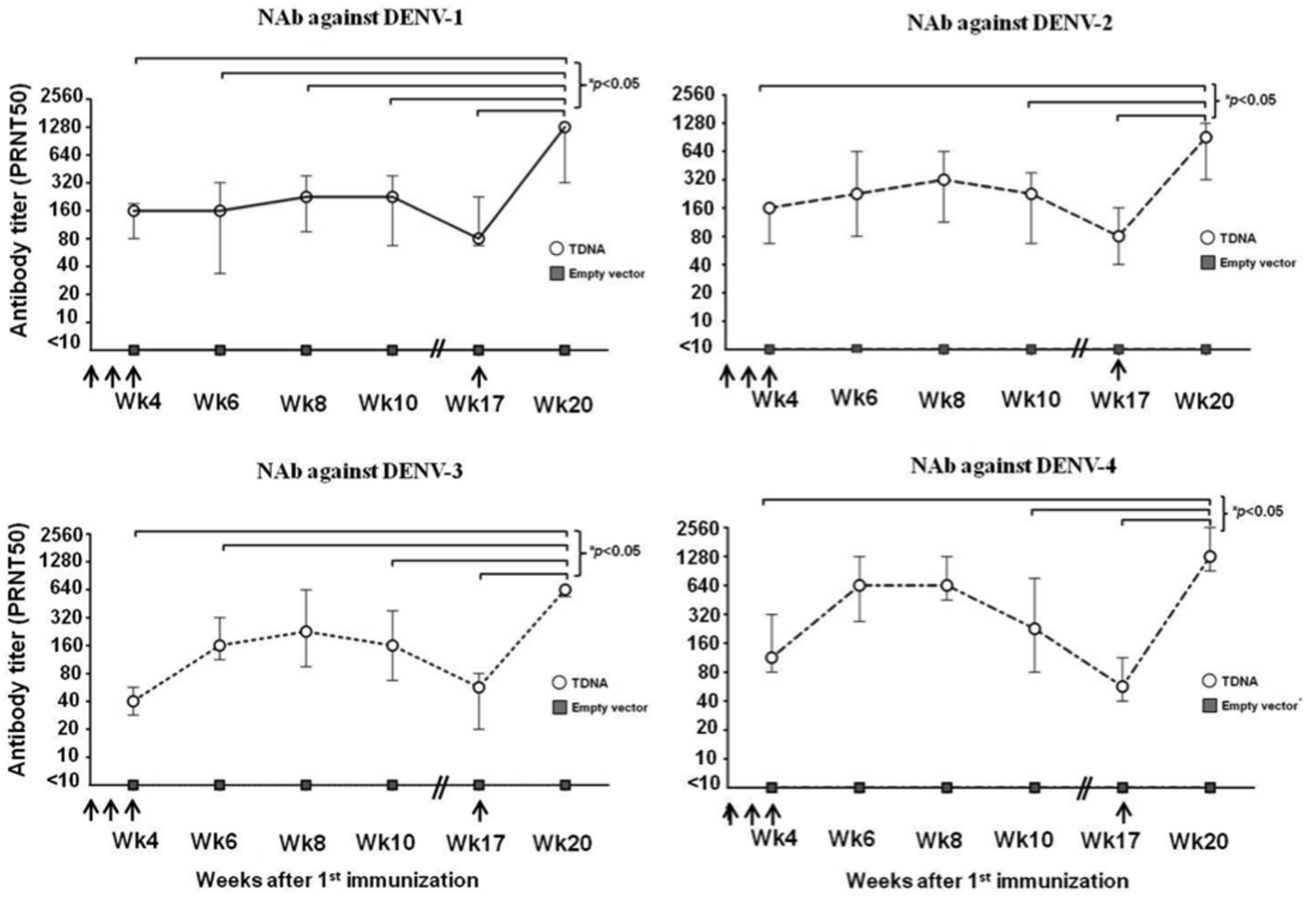
Kinetics of NAb responses following TDNA prime-boost immunization. NAb titers of individual mice sera (*n* = 6), against each of the four dengue serotypes were shown separately. Opened circle and grey square represent the median PRNT50 titer with inter-quartile ranges for the TDNA group and the empty vector group, respectively. Arrows represent the injection of each dose of TDNA. * indicates *p*<0.05.

In mice that were immunized for three times with 100 µg/dose of TDNA, NAb response were detected against all DENV serotypes at week 2 after the second immunization, reaching the peak levels at week 4 after the third dose (Figure 3). The NAb gradually declined over time but remained detectable at week 13 after the third dose. An additional TDNA boost injected at 3 months after the third dose resulted in a significant increase of the NAb titers (Figure 3). The median NAb titers increased by 16-fold (*p* = 0.012), 12-fold (*p* = 0.005), 10.7-fold (*p* = 0.005) and 21-fold (*p* = 0.007) against DENV-1, DENV-2, DENV-3, and DENV-4, respectively, when compared with the baseline levels at week 13 after the third dose.

## Discussion

In this study, the recombinant dengue PrME DNA vaccine candidates when immunized by intramuscular electroporation (IM-EP) as a tetravalent cocktail, generated good neutralizing antibody against all serotypes of DENV. With DNA/DNA prime/boost approach, the median NAb titers after the 4^th^ TDNA immunization at 1280, 640, 640 and 1280 against DENV-1, -2, -3 and -4, respectively. Our study confirms there is no interference observed when TDNA was administered in mice [5]. The serotype-specific responses was preliminary evaluated in monovalent regimen by using spring-power needle-free injector, the results showed that monovalent vaccinated mice show the highest NAb titers against their homologous serotypes with PRNT50 titers 160–320 after 3^rd^ injection. Cross-neutralization against other serotypes were at least four-fold lower than its homologous serotype, PRNT50 titers were 10–40 (unpublished data). We also demonstrated the potential of IM-EP in DNA vaccine dose reduction. Ten microgram of TDNA (2.5 µg/monovalent vaccine) generated the similar levels median NAb titers against all DENV when compared with the 100 µg TDNA dose. Whether such an efficient delivery system can significantly reduce the cost of DNA vaccine and can increase the accessibility of dengue vaccine in most resource-limited countries and warrants further investigation.

The dengue-specific NAb induced by this TDNA vaccine candidate was detectable at least up to approximately 3 months from the last immunization. The other study demonstrated that NAb induced by DNA vaccine encoding prME can persist until 30 weeks [5]. In addition, our study demonstrated that homologous DNA vaccine itself could be efficiently used as a boosting vaccine. This approach has been shown to be promising in a cancer DNA vaccine regimen [25]. It may be explained by previous observations that DNA vaccine is capable of induction both memory B and helper T cells [26], [27]. Further study is warranted in non-human primate and thereafter in clinical study whether this tetravalent dengue DNA vaccine can really be used in a sequential boosting regimen i.e. before a rainy season in order to enhance the recall the memory cells and NAb responses similar to the seasonal influenza vaccine approach [28]–[31].

Nonetheless, the magnitudes of NAb responses cannot be compared between studies due to the difference in the neutralizing antibody assay methodologies and reported unit. Ramanathan et al. reported the range of NAb responses of 400–1000 unit/ml when mice immunized with 10 µg of synthetic consensus EDIII by using IM-EP [32]. Konishi et al. reported PRNT70 NAb titers of 40–80 in mice after 2 doses of 100 µg tetravalent DNA immunization by needle-free injector [5]. In this study, the DNA/DNA prime/boost strategy induced median NAb titers at 1280, 640, 640 and 1280 against DENV1, 2, 3 and 4, after the 4^th^ TDNA immunization respectively. The induced NAb levels in this study were promising when compared to the previous report which showed protective efficacy in animal after viral challenge [33]. A recent study by Porter et al. demonstrated that tetravalent prME DNA formulated with Vaxfectin adjuvant with NAb titers before challenge against DENV-2 at 105–390 significantly reduced viremia compared to control group [34] and this vaccine formulation is ongoing in phase I trial.

Recent evidences have raised the relevant of dengue viral strains to be used in vaccine design and PRNT-50 assay. A concern in matching between the strain used for constructing a vaccine and the recent circulating virus has been raised from a recent phase 2b study conducted in Thailand [35]. A very low protection rate against DENV-2 (9.2%) was observed. The authors hypothesized that the mismatch between the DENV-2 CYD vaccine and circulating DENV-2 might be the cause of failure to protect against DENV-2 infection. In addition, the impact of more recent versus old dengue viral isolates on the PRNT50 results has also been observed by Thaisomboonsuk et al. [36]. They found that when DENV-4 strain 1036 (genotype-2, isolated in 1976, Indonesia) was used to test with DENV-infected serum samples collected during the 2005-2006 endemic season, the detected PRNT50 titers were less than expected. In contrast, the titers increased by 4.2 folds when a more recent Thailand DENV-4 isolate strain C0036 (genotype-1, isolates in 2006, Thailand) was used.

Interestingly, our observation on dengue virus serotype 4 has further supported the critical consideration of viral strain to be used for vaccine design and for PRNT50 assay while developing a vaccine. The synthesized consensus dengue *prME* DNA sequence used in our candidate TDNA vaccine were based on the sequences of dengue viral isolates reported to the GenBank up to 2003, whereas the old DENV-4 strain 1036 isolated from Indonesia in 1976 was used to test the post-immunized murine sera. The PRNT50 titers were found to be very low; but a significantly increase of the titer in approaching to those other serotype NAb titers when the newer viral isolates strain C0036 (Thailand 2006) was used for the assay (see Figure 2). To investigate how close of our candidate DNA vaccine to the recent dengue isolates is, analyses the homology of E protein amino acid sequence were performed between the D4prME vaccine construct and DENV-4 strain 1036 or strain C0036. The D4prME in the DNA vaccine is only 96.8% (479/495 amino acid) homology to strain 1036 (Indonesia 1976), in contrast it is 99.2% (491/495 amino acid) homology to strain C0036 (Thailand 2006). The 16 amino acids difference within E protein between the D4prME vaccine construct and DENV-4 strain 1036 might be sufficient to alter the neutralization capacity of NAb, of which requires further investigation. This finding supports the use of most recent dengue viral strain for both vaccine immunogen design and neutralization assays to avoid such mismatching outcomes. However, this may affect the use of such vaccine in other geographical areas.

Taken together, this study has shown that the tetravalent dengue prME DNA vaccine, with a very high homology DNA sequence to recent dengue viral isolates, induced good neutralizing antibody responses in mice; and the tetravalent dengue DNA/DNA prime/boost strategy is promising and is currently being evaluated in non-human primates.
